# A191 REAL WORLD USE OF ADALIMUMAB BIOSIMILAR AMGEVITA® (ABP-501) IN PATIENTS WITH INFLAMMATORY BOWEL DISEASE IN CANADA

**DOI:** 10.1093/jcag/gwae059.191

**Published:** 2025-02-10

**Authors:** M Chohan, R J Wani, J Copeman, D Martinez, R Lukanova, F Dawod, O Mooney

**Affiliations:** Amgen Canada Inc, Mississauga, ON, Canada; Amgen Canada Inc, Mississauga, ON, Canada; Amgen Canada Inc, Mississauga, ON, Canada; Amgen Canada Inc, Mississauga, ON, Canada; Adelphi Real World, Bollington, Cheshire East, United Kingdom; Adelphi Real World, Bollington, Cheshire East, United Kingdom; Adelphi Real World, Bollington, Cheshire East, United Kingdom

## Abstract

**Background:**

ABP-501 (AMGEVITA®), biosimilar to adalimumab reference product (RP), was approved by Health Canada in 2021 for use in different immune diseases, including inflammatory bowel disease (IBD), comprising Crohn’s disease (CD) and ulcerative colitis (UC).

**Aims:**

To assess the real-world use, effectiveness and satisfaction of ABP-501 in adalimumab naïve and switch patients with IBD across Canada.

**Methods:**

Data were drawn from the Adelphi IBD Disease Specific Programme^TM^, a cross-sectional survey with retrospective data collection from physicians conducted in Canada between July 2023 and June 2024. Gastroenterologists (GIs) provided data on their patients with IBD including demographics, clinical characteristics, and treatment satisfaction. Analyses were descriptive.

**Results:**

Overall, 10 GIs reported data on 101 patients (60 CD and 41 UC): 80 initiated ABP-501 as first adalimumab therapy (initiators) and 21 switched directly to ABP-501 from RP (switchers) (Table 1).

Mean [standard deviation; SD] time receiving ABP-501 at consultation was 13.2 [11.6] and 21.6 [9.9] months for initiators and switchers, respectively. Switchers received RP for 48 [27.1] months before switching to ABP-501. Most patients were receiving adalimumab as their first advanced therapy (70% initiators, 90% switchers). The main reasons for switching from RP to ABP-501 were cost effectiveness for the healthcare system (57%) and insurance coverage (43%).

At consultation, most initiators and switchers had mild disease (92%, 95%, respectively), were in clinical (91%; 95%, respectively) and endoscopic (66%; 81% respectively) remission (Figure 1) and had an improving/stable disease (97%; 100%, respectively). Treatment targets for initiators and switchers included maintaining clinical remission (n=54; n=18, achieved by 100%, respectively) and maintaining endoscopic remission (n=37; n=17, achieved by 86% and 100%, respectively). GIs were satisfied with ABP-501 treatment (99% initiators, 100% switchers).

**Conclusions:**

Most patients with IBD treated with ABP-501 as initiating or continuing adalimumab therapy in a real-world setting had mild disease, were in remission and had an improving/stable disease. Patients who switched from RP to ABP-501 switched mainly for economic reasons. GIs reported high levels of satisfaction.

Table 1. Demographics and clinical characteristics of patients with IBD receiving ABP-501 at consultation

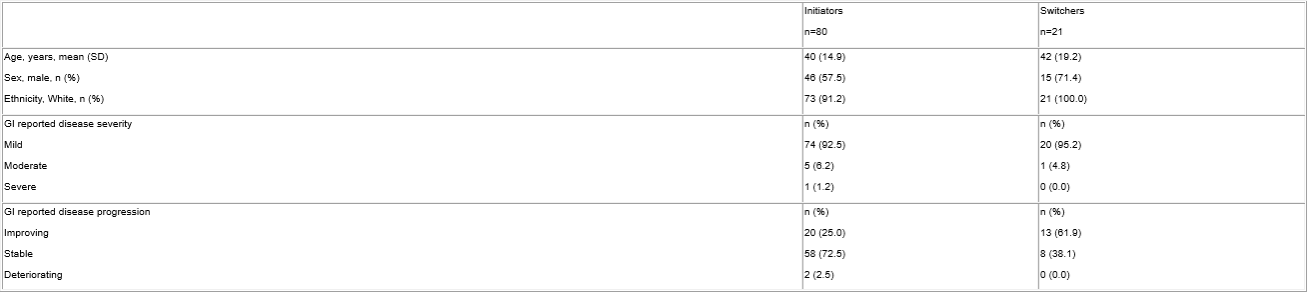

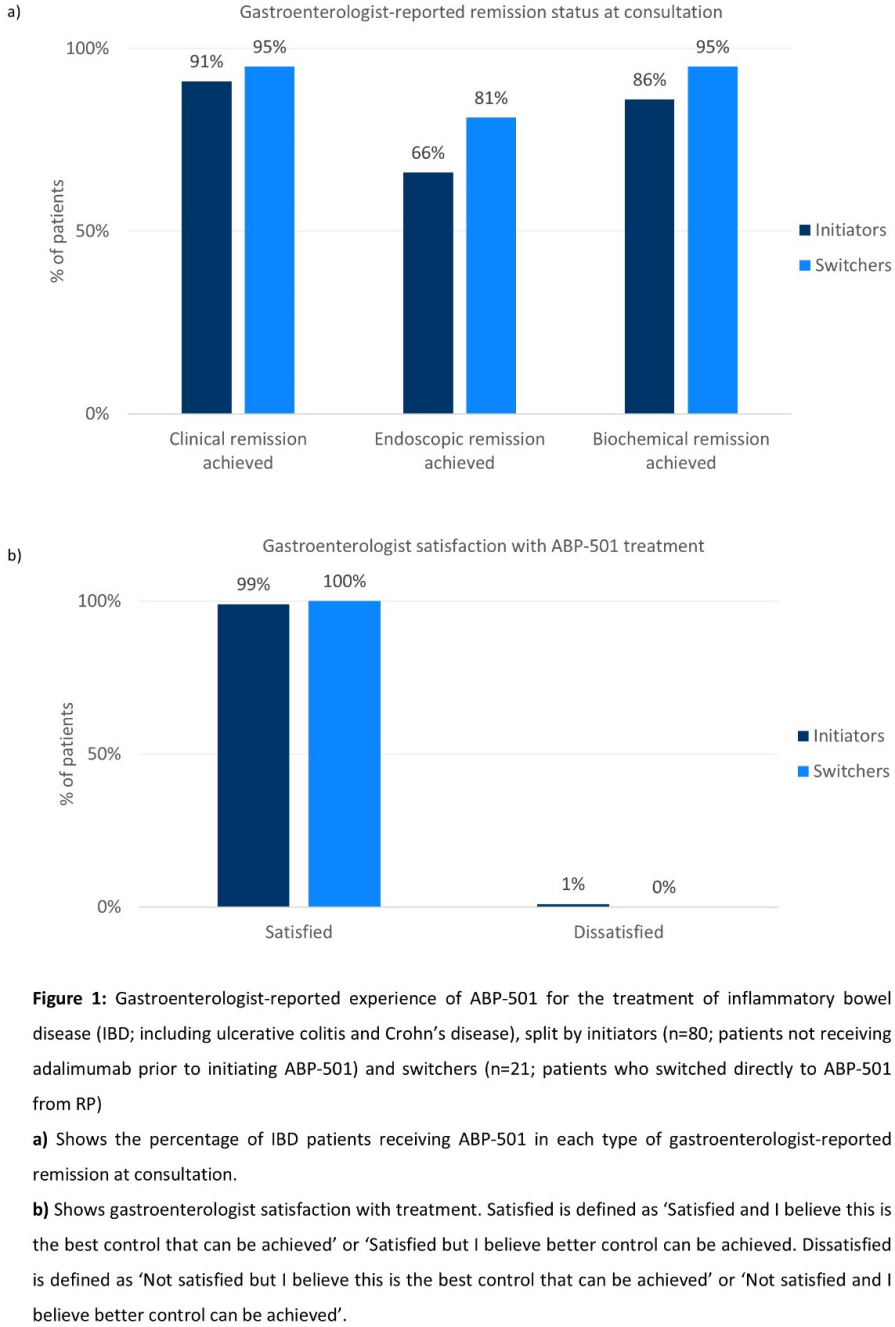

**Funding Agencies:**

None

